# Both sexes develop DKD in the CD1 uninephrectomized streptozotocin mouse model

**DOI:** 10.1038/s41598-023-42670-5

**Published:** 2023-10-03

**Authors:** Jackie Trink, Ifeanyi Kennedy Nmecha, Dan Zhang, Melissa MacDonald, Bo Gao, Joan C. Krepinsky

**Affiliations:** Division of Nephrology, St. Joseph’s Hospital, McMaster University, 50 Charlton Ave East, Rm T3311, Hamilton, ON L8N 4A6 Canada

**Keywords:** Kidney diseases, Experimental models of disease

## Abstract

Diabetic kidney disease (DKD) is characterized by a progressive increase in albuminuria and typical pathologic features. Recent studies have shown that sex is an important factor to consider in the pathogenesis of DKD. Presently, the hallmarks of this disease have primarily been studied in male rodent models. Here we explored the influence of sex in a murine model of DKD. CD1 mice underwent a right nephrectomy followed by intraperitoneal injection with 200 mg/kg streptozotocin to induce type 1 diabetes. Due to a high mortality rate, females required a reduction in streptozotocin to 150 mg/kg. Mice were followed for 12 weeks. Both sexes developed comparable hyperglycemia, while albuminuria and glomerular volume were increased to a greater degree in females and kidney hypertrophy was only seen in females. Males had a greater increase in blood pressure and glomerular basement membrane thickening, and a greater decrease in endpoint weight. Serum TGFβ1 levels were increased only in females. However, both sexes showed a similar increase in induction of kidney fibrosis. T cell and macrophage infiltration were also increased in both sexes. While some differences were observed, overall, both sexes developed clinical and pathologic characteristics of early DKD. Future studies evaluating therapeutic interventions can thus be assessed in both sexes of this DKD model.

## Introduction

The global prevalence of diabetes mellitus has increased more than fourfold from 108 to 537 million between the years 1980 and 2021^1,2^. Diabetic kidney disease (DKD) is a common complication, developing in up to 40% of patients^2^. DKD onset is generally characterized by the development of albuminuria, glomerular hypertrophy, expansion of the glomerular mesangium, thickening of the glomerular basement membrane, and tubular atrophy. Progressive DKD leads to end stage kidney disease requiring dialysis or a kidney transplant^3–5^. Current standard of care consists of a multifactorial approach including blood glucose and blood pressure control, and the use of renin–angiotensin–aldosterone system (RAAS) inhibitors. More recently, sodium-glucose cotransporter-2 (SGLT2) inhibitors were also shown to be effective in type 2 DKD patients. Unfortunately, these therapeutic interventions are unable to prevent the progression of DKD. Elucidating the mechanism(s) underlying DKD and testing novel therapeutics in appropriate preclinical models is thus of high clinical importance.

Sex is an important biological factor that plays a diverse role in physiology and pathology. Men with type 2 diabetes in general progress more quickly to end stage DKD compared to women, thus requiring more frequent kidney replacement, although studies in older females have shown faster rates of progression associated with increased proteinuria compared to men^6^. This may be due to an older age of onset and thus decreased circulating estrogen and progesterone levels, although effects of hormone replacement therapy on kidney function have been inconsistent^7^. Furthermore, it is now recognized that non-albuminuric DKD may also be seen, with this phenotype more likely to develop in females^8^. These differences may influence treatment decisions and response, highlighting the importance of including both male and female participants in clinical studies.

The effects of sex and sex hormones on DKD have also been studied in preclinical models. In rat models of type 1 or type 2 diabetes, DKD was more pronounced in males, with greater albuminuria, blood pressure and fibrosis, and with lower kidney function associated with decreased nitric oxide, respectively^9,10^. Interestingly, conflicting data exist in preclinical mouse models, with comparable onset of DKD in male and female diabetic mice in some models^11,12^, but more advanced DKD in females in type 1 diabetic OVE26 mice^13^. In the latter, diabetes was associated with lower plasma estradiol and renal expression of estrogen receptors, suggesting a potential protective effect of estrogen in the development and progression of DKD. Indeed, administration of estradiol was shown to decrease angiotensin II binding to its type I receptor in the kidney^14^, an effect that would be expected to confer protection against DKD. Conversely, castration worsened DKD in males, but this was reversed only by lower doses of testosterone administration, with higher doses worsening the phenotype^15^. Thus, differences in hormone levels lead to variability in disease manifestation, severity, and progression in preclinical models, emphasizing the need to include both males and females in preclinical mechanistic and treatment studies.

Historically, preclinical DKD studies have predominantly used male mice. More recently, efforts at characterizing DKD in both sexes in rodent models are emerging to promote the incorporation of female mice into therapeutic studies^16–18^. A commonly used preclinical model of DKD is the induction of type 1 diabetes with streptozotocin (STZ) in CD1 mice after uninephrectomy. Diabetes in this strain induces more marked kidney fibrosis in comparison to either 129SV or commonly used C57BL/6 mice^19^. Studies in other mouse strains have shown that female mice are relatively resistant to developing hyperglycemia after STZ administration compared to males^20^. However, the effect of sex in CD1 mice on the development of diabetes or DKD have not as yet been assessed and are the focus of this study.

## Results

### Both sexes developed hyperglycemia, with female mice more sensitive to STZ effects

**Figure 1:**
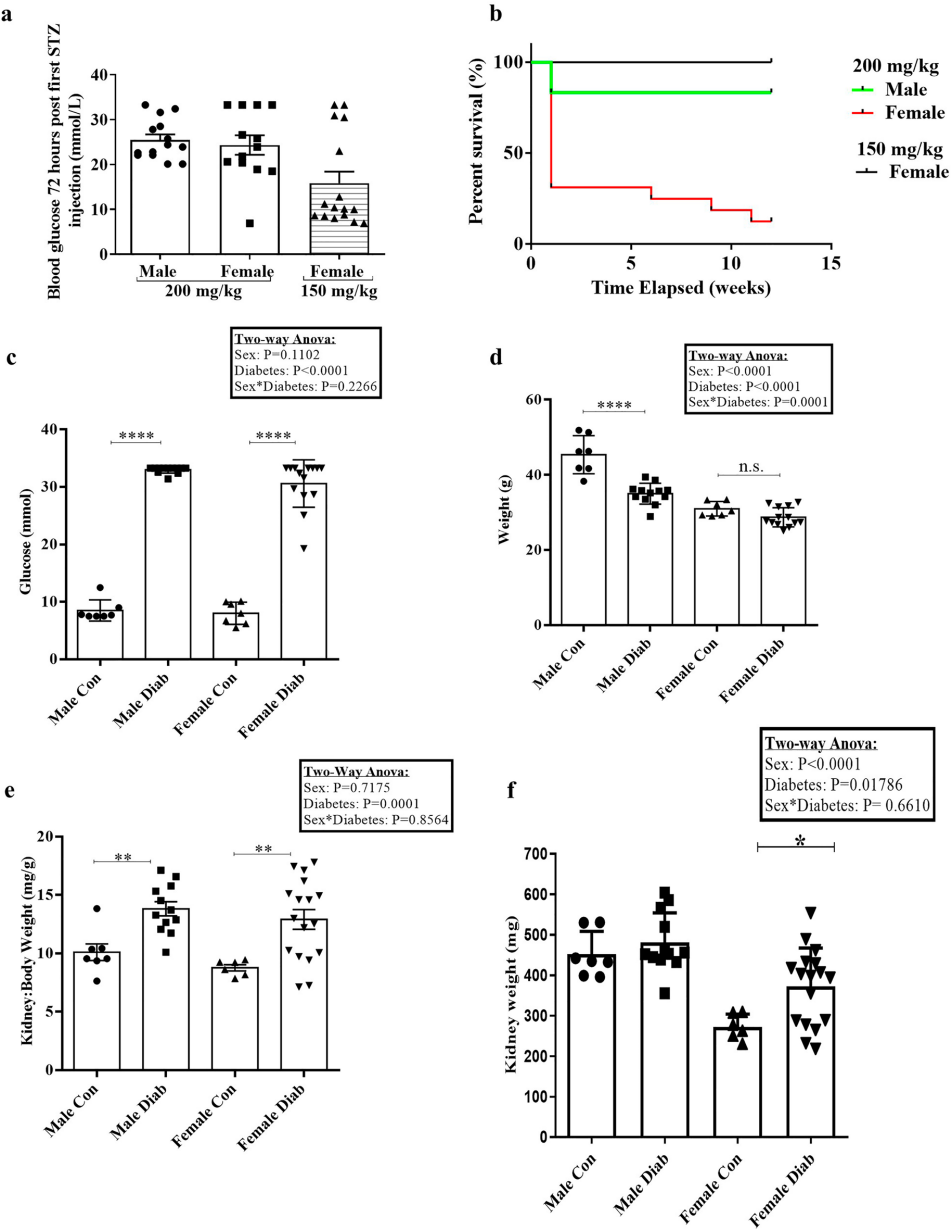
Clinical effects of STZ in male and female mice. (**a**) Blood glucose levels (mmol/L) in male and female mice 72 h after first STZ administration at the indicated doses (n = 14-16). (**b**) Survival curves in mice after receiving STZ. (**c**) Blood glucose at 12 weeks was increased after STZ in both male and female diabetic mice, but female mice had more variability than males (****p < 0.0001) (n = 7–15). (**d**) Weight reduction at end-point was seen only in males (****p < 0.0001, *p *= 0.0564) (n = 7–14). (**e**) Kidney-to-body weight ratio was increased in both sexes with diabetes, suggesting kidney hypertrophy (**p < 0.01, ****p < 0.0001) (n = 7–14). However, when kidney weight alone was considered (**f**),  significant kidney hypertrophy was only seen in female mice (*p<0.05)(n=7-17).

To induce diabetes, mice initially received a single intraperitoneal 200 mg/kg STZ injection. All male mice developed hyperglycemia within 72 h (Fig. 1a). However, this dose caused death in 11/16 female mice within 1 week, with another 3 deaths at 6, 9 and 11 weeks. Thus, only two of this cohort survived to 12 weeks, with a total mortality of 14/16, or 87.5%. This is in comparison to male mice, in which 2/14 (14.3%) of mice died. To induce diabetes in females, STZ dose was thus reduced in subsequent cohorts to 150 mg/kg, resulting in 100% survival to 12 weeks (Fig. 1b), but necessitating reinjection in 10/16 (62.5%) mice 1 week after initial injection. STZ dose was reduced to 100 mg/kg for reinjection. Although glucose was somewhat more variable than in males, this dosing resulted in 12 of 16 female mice (75%) reaching the enrollment blood glucose of  > 17 mM.

Mice were followed for 12 weeks after confirmation of diabetes. Endpoint blood glucose levels in mice were measured prior to sacrifice. There was no difference between sexes in control mice. Although both sexes were significantly hyperglycemic at 12 weeks, more variability in serum glucose was seen in female mice (Fig. 1c). Overall, a 2-way analysis of variance (ANOVA) did not indicate a sex effect on hyperglycemia.

### Weight, kidney hypertrophy, and blood pressure

Endpoint weight was reduced in male diabetics compared to controls. Although female mice had lower weight than males, diabetes did not induce significant weight loss as was seen in males (Fig. 1d). Both male and female diabetic mice exhibited an increase in kidney-to-body weight ratio (Fig. 1e). However, since only males showed significant weight loss with diabetes, we also considered kidney weight alone. As shown in Fig. 1f, kidney weight was only significantly increased in females. Blood pressure was measured at 4, 8, and 12 weeks after enrollment into the study and analysed by repeated measures 3-way ANOVA.  As seen in Table 1, blood pressure was elevated in both male and female mice at different time points. At 12 weeks, male diabetic mice had higher diastolic blood pressure than female diabetics.

**Table 1:** Blood pressures.

	Male control	Male diabetic	Female control	Female diabetic
4-week SBP (mmHg)	121 ± 2	139 ± 5**	123 ± 4	146 ± 7*
4-week DBP (mmHg)	98 ± 2	115 ± 5*	100 ± 3	117 ± 7
8-week SBP (mmHg)	119 ± 3	157 ± 4****^,+^	119 ± 5	142 ± 5**
8-week DBP (mmHg)	105 ± 6	130 ± 4**^,+^	96 ± 5	112 ± 5
12-week SBP (mmHg)	126 ± 6	156 ± 5**^,+^	120 ± 2	138 ± 6*
12-week DBP (mmHg)	103 ± 6	129 ± 5**^,&^	98 ± 3	104 ± 5

*SBP* Systolic blood pressure, *DBP* Diastolic blood pressure, data presented as mean ± SD.

Significance between control and diabetic groups are represented as *p < 0.05, **p < 0.01, ***p < 0.0001.

Significance between male and female diabetic groups are represented as ^+^*p *< 0.05, ^&^*p *< 0.01. By repeated measures 3-way ANOVA: SBP: Only the effect of diabetes was significant (p<0.0001), with interaction between time*sex p=0.07. DBP: The effects of sex (p=0.04), effect of diabetes (p<0.0001), and interaction between time*sex (p=0.2) were significant.

### Albumin to creatinine ratio and TGFβ1 secretion differed between male and female diabetic mice

**Figure 2:**
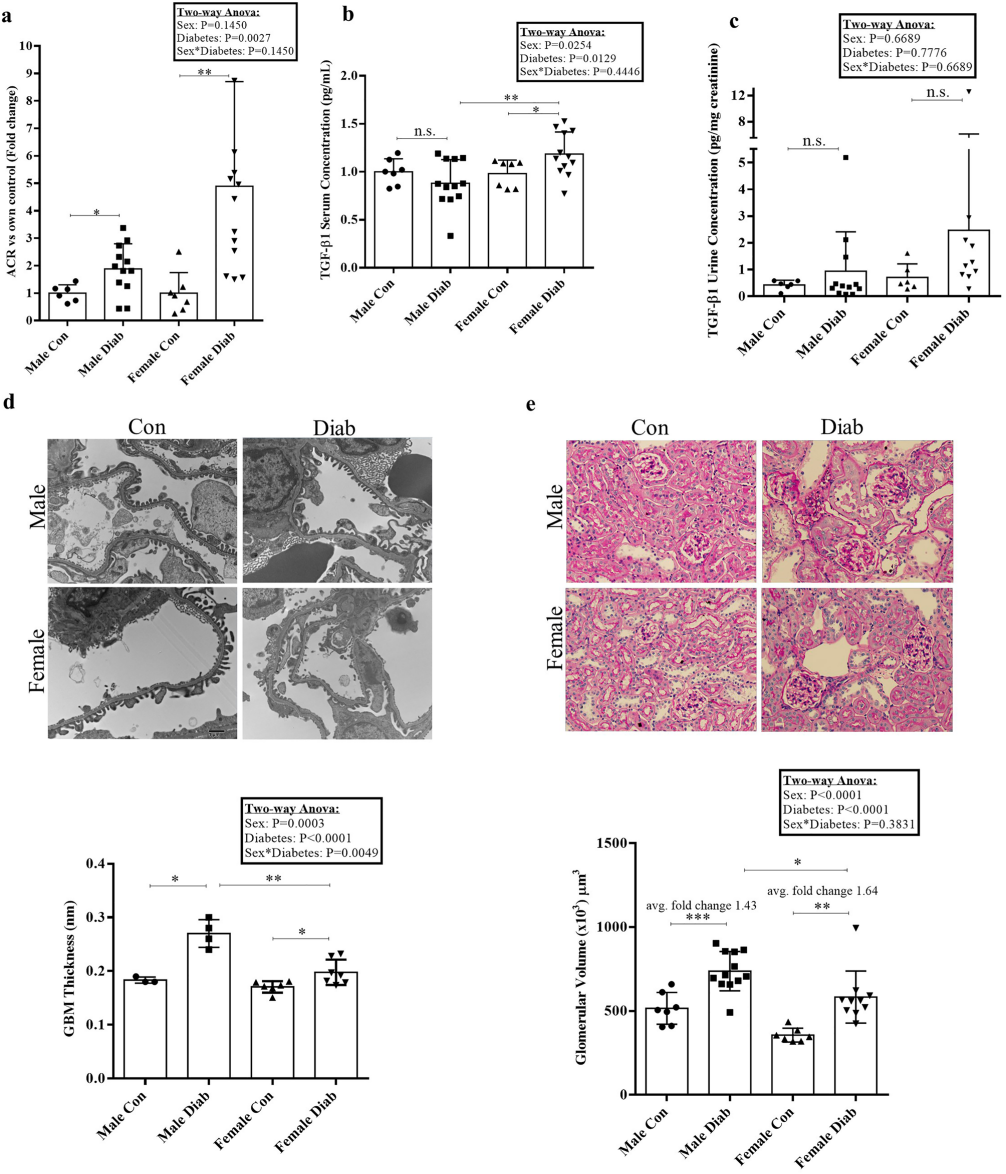
Albuminuria, TGFβ1 and early pathologic changes. (**a**) Urine albumin to creatinine ratio was increased in all diabetic mice, but higher in female compared to male diabetics (*p = 0.04 between male groups, *p = 0.02 between female groups, *p = 0.014 between diabetic groups) (n = 6–14). (**b**) TGFβ1 serum levels were only increased in female diabetic mice, (Mann-Whitney U test, *p *= 0.26 between male groups, *p = 0.05, **p = 0.01) (n = 7–12). (**c**) Urine TGFβ1 levels were increased in both male and female diabetic groups, but this did not reach significance (Mann-Whitney U test, *p *= 0.41 between male groups and *p *= 0.26 between female groups) (n = 6–12). (**d**) Glomerular basement membrane (GBM) thickening was seen to a greater degree in male compared with female diabetics (**p = 0.003 between male groups, *p = 0.026 between female groups, **p = 0.001 between diabetic groups) (n = 3–7). (**e**) Although glomerular volume was greater overall in male mice, the increase seen with diabetes was higher in female mice (by fold change) (***p = 0.0005, **p = 0.002, *p = 0.015) (n = 7–12).

We observed a marked increase in albuminuria in all diabetic mice compared to wild-type controls, with a greater increase in female diabetics (Fig. 2a). The profibrotic cytokine TGFβ1 is an important mediator of fibrosis in animal models and human DKD^21^. We thus measured total TGFβ1 levels in the serum and urine. Serum TGFβ1 was increased only in female diabetic mice (Fig. 2b). While an increase in urinary TGFβ1 was also seen in male and female diabetic mice, this was not statistically significant (Fig. 2c).

### Early glomerular pathology and accumulation of profibrotic proteins

Using electron microscopy, we measured the thickness of the glomerular basement membrane (GBM), which is characteristically increased in early DKD^3^. Here we observed an increase in GBM thickness in both male and female diabetic mice, with male mice exhibiting a greater increase and 2-way ANOVA showing interaction between sex and diabetes (Fig. 2d). An increase in glomerular volume is also seen in the early stages of DKD^3^. Figure 2e shows that glomerular volume was increased in both diabetic groups, but the relative increase was greater in females (1.64 fold change) compared to males (1.43 fold change).

**Figure 3:**
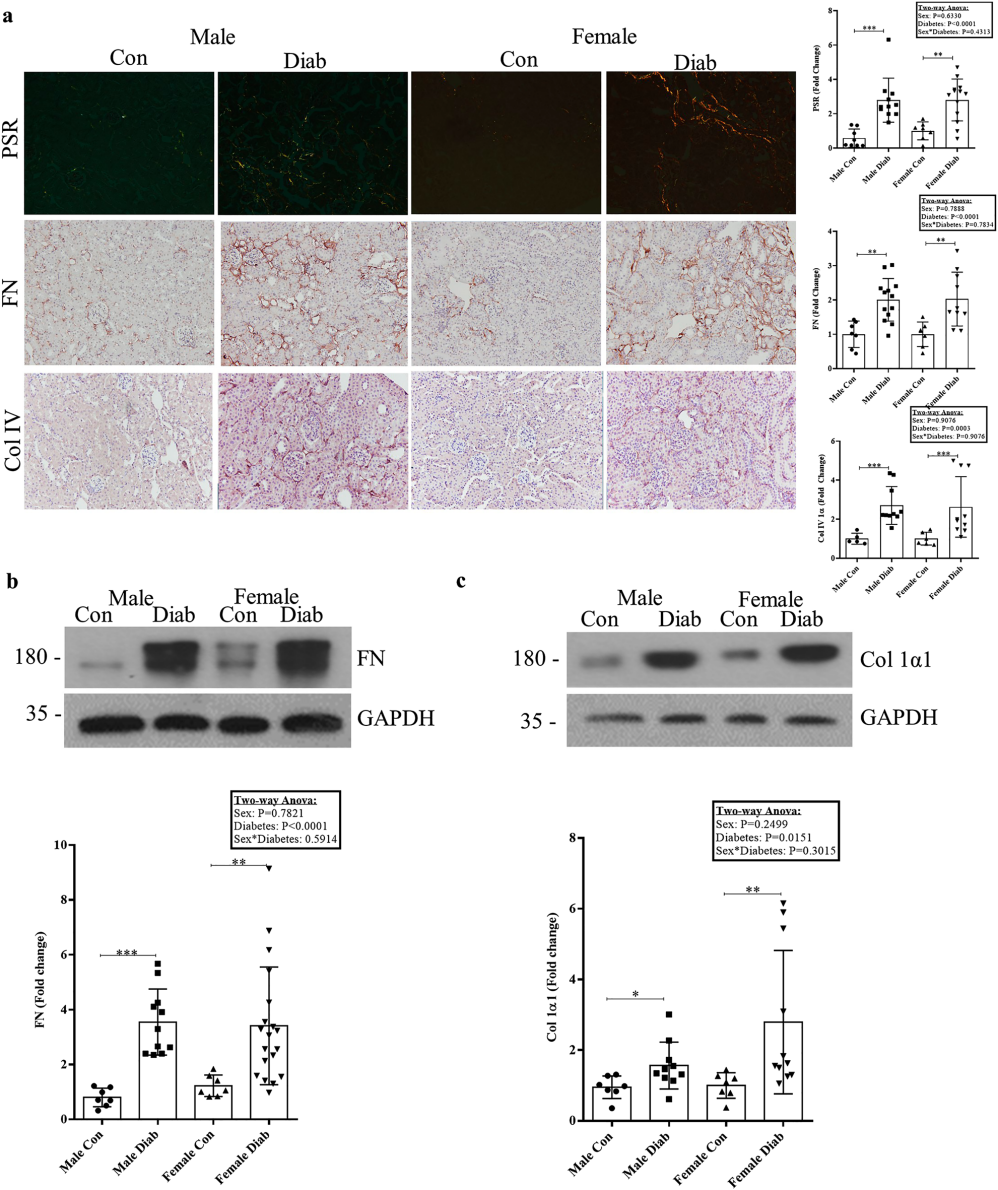
Accumulation of profibrotic proteins in diabetic kidneys. (**a**) Staining for collagens I/III by PSR (Mann-Whitney U test, ***p = 0.0003, **p = 0.002) (n = 8–13), fibronectin (FN) (**p = 0.001 between male groups, **p = 0.006 between female groups) (n = 7–13) and collagen IV (Mann-Whitney U test, **p = 0.002, *p = 0.025) (n = 5–10) by IHC showed comparable increases between male and female diabetic mice. Immunoblotting of kidney lysates for (**b**) FN (Mann-Whitney U test, ***p = 0.0004, *p = 0.048) (n = 7–19) and (**c**) Col 1α1 (Mann-Whitney U test, *p = 0.039 between male groups, *p = 0.036 between female groups) (n = 7–11) show similar increases in diabetic male and female mice.

We next assessed known extracellular matrix proteins relevant to the fibrotic phenotype seen in DKD. We stained for collagens I and III using picrosirius red (PSR) and collagen IV (Col IV) as well as fibronectin (FN) by IHC. Figure 3a shows that both male and female diabetic mice exhibited a marked increase in the staining of all matrix proteins, with no observable difference between males and females. Similarly, Fig. 3b,c immunoblotting for FN and collagen 1α1 (Col 1α1) show an increase in both matrix proteins in diabetic mice, with no difference between sexes.

### T cell and macrophage infiltration are similar between sexes

**Figure 4:**
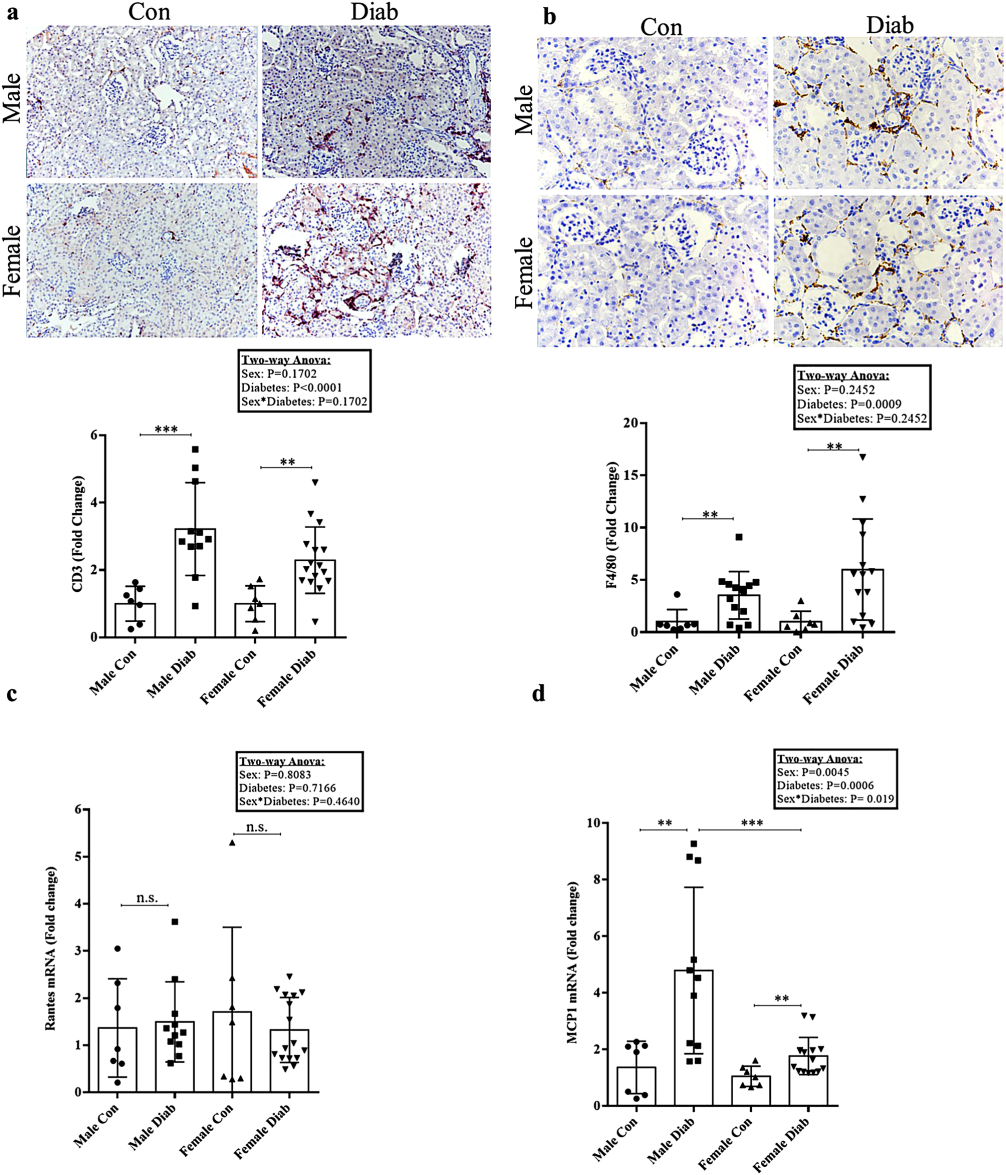
T cell and macrophage infiltration in diabetic kidneys. IHC for (**a**) T cell infiltration (CD3) (***p = 0.0009, **p = 0.003) (n = 7–16) and (**b**) macrophage infiltration (F4/80) (*p = 0.013 between male groups, **p = 0.005 between female groups) (n = 7–14) show marked increases in both male and female diabetic kidneys. T cell and macrophage infiltration were comparable between sexes. (**c**) RT-PCR for Rantes, a chemokine involved in T cell infiltration, showed no increase in either diabetic group (*p *= 0.777 between male groups, *p *= 0.509 between female groups) (n = 7–16). (**d**) RT-PCR for MCP1, a chemokine involved in macrophage infiltration, was increased to a greater extent in male compared to female diabetic kidneys (Mann-Whitney U test, **p = 0.009 between male groups, *p = 0.017 between female groups, **p = 0.002 between diabetic groups) (n = 7–15).

Inflammation, with increases in both T cells and macrophages, is also a characteristic finding in DKD^17,22^. We thus assessed whether there was any difference in inflammation in DKD between male and female mice. Using IHC, we observed an increase in the expression of CD3 (Fig. 4a), a marker for T cell infiltration^23^, as well as an increase in F4/80 (Fig. 4b), a marker for activated macrophages^17^, in both male and female diabetic mice. We next assessed the transcript expression of chemokines known to be increased in DKD and to recruit inflammatory cells. While no difference was found in the transcript levels for RANTES/CCL5, a chemoattractant for T cells^22^ (Fig. 4c), a significant increase was seen in both sexes with diabetes for MCP1/CCL2, a chemoattractant for monocytes/macrophages^22^ (Fig. 4d). MCP1 was, however, markedly increased in male diabetics compared to female diabetics. Both individual effects of sex and diabetes and their interaction were seen.

## Discussion

Sex differences exist in the susceptibility for development and progression of DKD, as well as in response to established therapies^16^. This, and the growing prevalence of DKD, support the need for assessing both sexes in preclinical studies. In this study, we compared DKD in uninephrectomized male and female outbred CD1 mice in which diabetes was induced by high-dose STZ. Prior studies have established this as a model of early human DKD in male mice, characterized by hypertrophy, albuminuria and both glomerular and tubulointerstitial fibrosis^19^. However, thus far only male mice have been characterized. In this study, we provide the first evidence that DKD is also seen in female CD1 mice, although some phenotypic differences exist between the two sexes. We also describe important differences in the use of STZ for model generation.

Females have not traditionally been included in DKD models, particularly those induced by STZ, due to their partial protection by the female hormone estrogen against STZ-induced pancreatic beta cell injury^24^. It was shown in inbred C57BL/6 J mice that low-dose STZ (40 mg/kg/day for 5 days) induced higher blood glucose in males throughout the 6-week observation period. Interestingly, insulin resistance also developed only in males, with estrogen having been found to additionally confer insulin sensitivity^24–26^. A comparison of 5 strains of inbred mice similarly showed that males consistently developed more robust hyperglycemia with 40 mg/kg/day STZ for 5 days (given at 8 and 15 weeks of age) than did females^20^. In eNOS knockout mice on a C57BL/6 background in which diabetes was induced by low-dose STZ, a sixth dose was required in female mice to induce equivalent hyperglycemia^11^. High-dose STZ is typically used in CD1 mice to induce type 1 diabetes. Our own data with this model show that male mice exhibited a more consistent increase in blood glucose levels with less variability after STZ injection compared to females, supporting an influence of sex in STZ susceptibility. Indeed, more male mice required implantation of an insulin pellet to maintain body weight and allow survival (7/14 or 50% for males vs 3/14 or 21.4% for females). This was reflected by a reduced endpoint weight in males that was not as markedly observed in females. These are similar findings to the low-dose STZ protocol in eNOS knockout mice^11^. Further, a reduction in STZ dosage from 200 to 150 mg/kg was required due to the initial high mortality rate in females. Once reduced, female survival was high, but more mice required reinjection to induce equivalent diabetes compared with males (none for males vs 10/16 or 63% for females), and fewer female mice met enrollment criteria. Thus, sexual dimorphisms were seen in the induction and maintenance of diabetes in CD1 mice.

The effect of sex on the development of diabetes has also been examined in non-STZ diabetes models. In the OVE26 model, in which calmodulin is overexpressed in pancreatic beta cells, leading to their loss and early onset of type 1 diabetes in FVB mice, females had lower glucose levels than males^13^. Conversely, no difference in hyperglycemia was seen between sexes in obese C57BLKS type 2 diabetic db/db mice, both with and without eNOS deficiency^12,27^. In a spontaneous nonobese rat model of type 2 diabetes, however, females had lower glucose and only the males displayed insulin resistance^28^. Given this variability, it is thus important to establish the effects of sex in any diabetic model used to conduct preclinical therapeutic trials. These differences also support the need to include both male and female cohorts in preclinical studies to account for any differences in the development and progression of diabetes and its complications that could be attributed to sex.

After equivalent establishment of diabetes in males and females, we observed overall that both sexes developed typical features of DKD. However, while development of fibrosis was similarly increased in both sexes, differences in the degree of change in other parameters were noted. Kidney hypertrophy as well as increased albuminuria was greater in females, but also somewhat more variable, while GBM thickening was more pronounced in males. Interestingly, while blood pressure increases were similar at 4 weeks, divergence was seen at later time points, with females showing less hypertension than males. Furthermore, TGFβ1 serum concentrations were increased only in female diabetic mice. Lane et al*.* showed that female mice had a threefold increase in TGFβ1 production after puberty, suggesting regulation by sex hormones^21^. This interaction between sex hormones and TGFβ1 may have led to the higher serum concentrations of TGFβ1 in females. Another possible explanation could be due to a higher proportion of diabetic female mice requiring an additional STZ dose due to insufficient induction of hyperglycemia with a single reduced dose of STZ (150 mg/kg). Given the greater variability that was seen in glucose levels in females, and the greater difficulty in inducing hyperglycemia in females, we further analysed key parameters in the subset of male and female mice whose glucose was >27mmol/L 72h after their first injection of STZ. This is shown in Supplementary Figure 1. In this subset, glucose was equivalent as expected based on inclusion (Supp. Fig. 1a). Kidney weight was signficantly increased by diabetes in both sexes (Supp. Fig. 1b). Although a difference was seen between male and female diabetic, with male diabetics having greater kidney weight, this was likely due to the higher baseline organ weight in males. ACR was increased in both males and females as previously seen, but in this subset the difference between male and female diabetics was significant (Supp. Fig. 1c). The differences previously found in the glomerular volume (Supp. Fig. 1d) and development of fibrosis as assessed by PSR and IHC for fibronectin and collagen IV were maintained. This suggests that albuminuria is more prominently induced by hyperglycemia in females, but otherwise key features of DKD are similar between the sexes.

Inflammation has been implicated in the pathogenesis of DKD, with increases in chemokines that regulate T cell and macrophage recruitment to the kidney^22,29,30^. Our data show significant increases in the number of infiltrating T cells and macrophages in male and female diabetic mice. Increased expression of the macrophage-recruiting chemokine MCP-1 at the transcript level was also seen in both male and female diabetic mice^31^, although higher levels were seen in males. Interestingly, we did not observe any difference in diabetic kidneys in the transcript expression of RANTES, the chemokine known to recruit T cells in DKD. As it is expected that the combined effect of multiple chemokines results in the recruitment of various inflammatory cell types, more extensive phenotyping would be required to characterize male and female diabetic kidneys comprehensively in this model. Overall, however, our data indicate that both male and female diabetic mice exhibit a similar pro-inflammatory phenotype characteristic of DKD.

In conclusion, we have identified a protocol to produce similar type 1 diabetes in male and female uninephrectomized CD1 mice with high dose STZ. Using this model, we have shown that DKD develops in both sexes. This enables future therapeutic studies to be conducted with both male and female cohorts.

## Methods

### Experimental animals

The total sample cohort consisted of 46 (14 male and 32 female) CD1 mice (Charles River Laboratories). All mice studies were conducted in accordance with McMaster University, the Canadian Council on Animal Care, and ARRIVE guidelines. All animal studies were approved by the McMaster University Animal Research Ethics Board (animal ethics protocol number: 18-07-30) and carried out in accordance with the principles of laboratory animal care by McMaster University and Canadian Council on Animal Care guidelines. To induce DKD, mice underwent a right nephrectomy. After 1 week of recovery, they were injected with streptozotocin (STZ) (200 mg/kg) after a 4 h fast. As STZ begins to degrade within minutes of buffer suspension^18^, for consistency mice were injected within 5 min of its reconstitution in sodium citrate buffer. Control mice were administered an equal volume of citrate buffer. During the first 48 hoursh post STZ injection, mice were given 10% glucose in their drinking water to prevent hypoglycemia. After 72 hours, blood glucose was measured using a glucometer on a blood sample from the tail vein and mice with values > 17mM were enrolled into the study. Mice differing from this protocol were discussed above in the “Results” section. Studies were done in sequential cohorts of mice to facilitate monitoring and harvest. Diabetic mice that developed ketonuria (dipstick, Bayer Multistix) were administered ¼ of an insulin pellet (LinShin Canada) to maintain body weight while also maintaining hyperglycemia. Blood pressure was measured at 4, 8, and 12 weeks using tail-cuff plethysmography (Coda non-invasive blood pressure monitoring system, Kent Scientific). After 12 weeks of diabetes, mice were anesthetized, perfused with saline and kidneys were harvested for further analysis.

### Immunohistochemistry, Masson’s Trichrome, and Picrosirius red staining

Formalin-fixed paraffin-embedded kidneys were cut at 4µm, deparaffinized and stained for trichrome (Sigma), PSR (Polysciences Inc.), or using the following antibodies: fibronectin (FN) (Sigma), collagen IV α1 (Col IV) (Novus), and CD3 (Dako). Antigen retrieval was used for FN (proteinase K, 40 µg), Col IV (citric acid steaming 30 min), and CD3 (citric acid steaming 30 min) staining prior to blocking and overnight primary antibody incubation. F4/80 staining was performed by the McMaster Immunology Research Centre. Images were captured using the Olympus BX41 microscope at 20 × and quantified using ImageJ software.

Periodic acid-Schiff (PAS) staining was completed on 4 µm paraffin-embedded kidney sections. Glomerular hypertrophy was assessed by measurement of glomerular cross-sectional area as described previously^32^.

### Measurement of albumin to creatinine ratio

At study endpoint, six-hour urine samples were collected using metabolic cages (Nalgene, 650-0210). Urinary albumin and creatinine were measured using Albuwell M (Exocell) and Mouse Creatinine Assay kits (Crystal Chem) respectively to determine the albumin to creatinine ratio (ACR, reported as µg/mg).

### Measurement of TGFβ1

Total TGFβ1 in serum or urine was measured using the TGFβ1 Quantikine ELISA Kit (R&D Systems) after acid activation of samples. Urine measurements were normalized to urine creatinine.

### Protein extraction and western blotting

Mouse kidney cortex samples were stored in liquid nitrogen. For protein extraction, kidneys were homogenized using 1.4mm ceramic beads (Lysing Matrix D, MP Biomedicals) in tissue lysis buffer with protease inhibitors (complete Mini, Sigma, and PhosSTOP, Sigma) using the Bead Mill Homogenizer (Bead Ruptor Elite, Omni International). Samples were then clarified, and protein concentration measured. Samples were separated using SDS-PAGE, then immunoblotted using the following antibodies: collagen I α1 (Col Iα1) (1:1,000, Cedarlane), fibronectin (FN) (1:1,000, Abcam), and GAPDH (1:1,000, Millipore).

### Statistical analysis

GraphPad Prism 6.0 was used to analyze differences between groups. Data points were assessed for outliers within each group using the Grubbs outlier test, and outliers were removed. Data points within groups were assessed for normality using Shapiro–Wilk normality test. If this was passed, data were further analyzed using a 2-way ANOVA to determine the main effects of sex and diabetes, and the interactions between them, with Tukey's Post Hoc test used or comparison within groups. However, if a group failed the normality test, data was analyzed using Mann–Whitney U test and this is indicated in the figure legend. Statistical significance was set at *p *≤ 0.05 and data are presented as mean ± SD. 

 Finally, repeated measures 3-way ANOVA was used to analyze blood pressure data to highlight any independent effect of time as a repeated factor. Pairwise comparison with Tukey's Post Hoc test was used to determine differences among the groups.

### Supplementary Information

Supplementary Figure 1.

Supplementary Figure 2.

## Data Availability

The data obtained and presented in this article are available from the corresponding author upon reasonable request.
