# Supplementary material for: Both sexes develop DKD in the CD1 uninephrectomized streptozotocin mouse model
Source: Sci Rep. 2023 Oct 3;13:16635. doi: 10.1038/s41598-023-42670-5 (PMC10547794; doi:10.1038/s41598-023-42670-5)
Supplement: Supplementary file 1 — Supplementary Figure 1. [file 41598_2023_42670_MOESM1_ESM.pptx]

## Slide 1
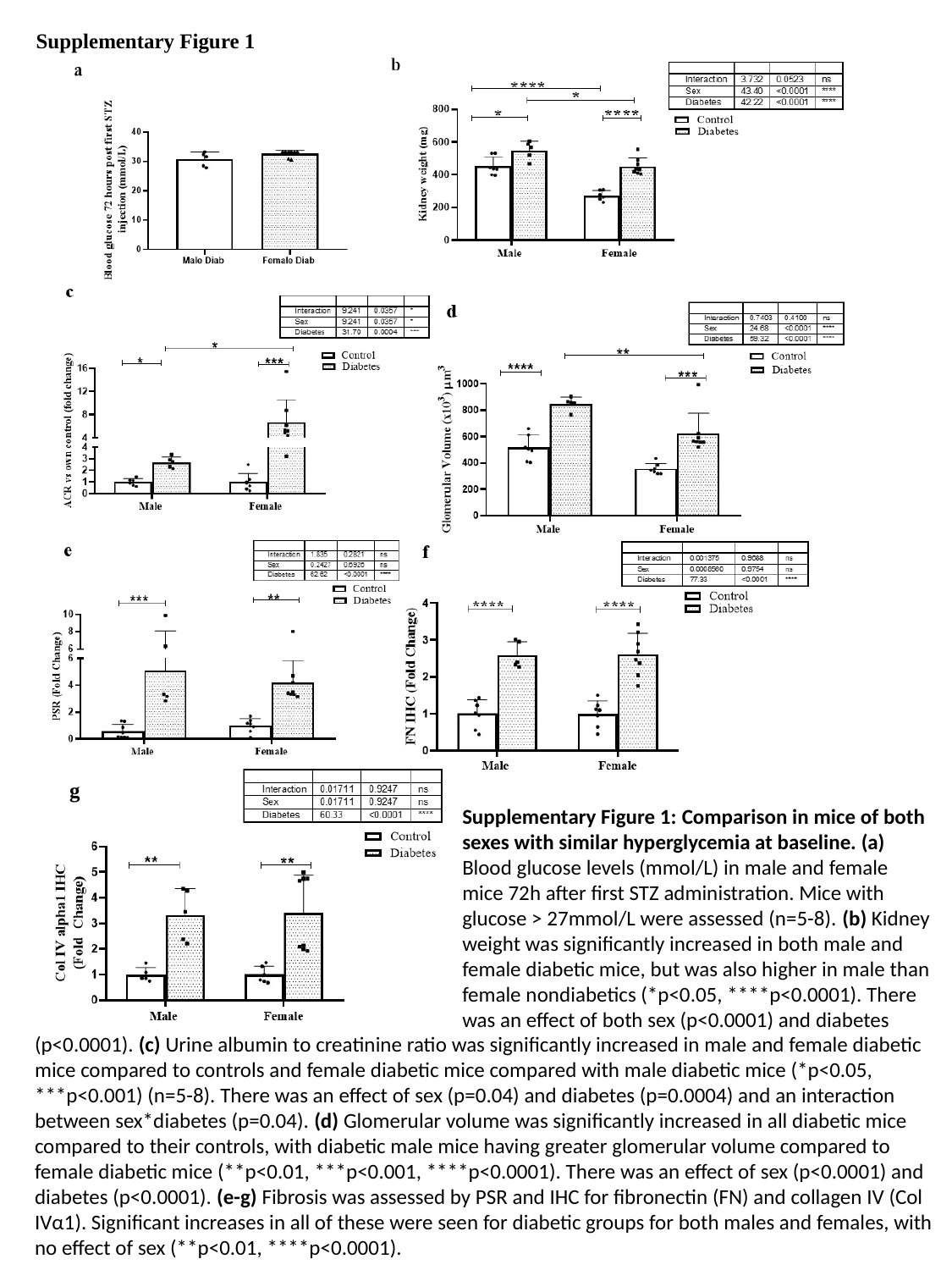

Supplementary Figure 1
g
Supplementary Figure 1: Comparison in mice of both sexes with similar hyperglycemia at baseline. (a) Blood glucose levels (mmol/L) in male and female mice 72h after first STZ administration. Mice with glucose > 27mmol/L were assessed (n=5-8). (b) Kidney weight was significantly increased in both male and female diabetic mice, but was also higher in male than female nondiabetics (*p<0.05, ****p<0.0001). There was an effect of both sex (p<0.0001) and diabetes
(p<0.0001). (c) Urine albumin to creatinine ratio was significantly increased in male and female diabetic mice compared to controls and female diabetic mice compared with male diabetic mice (*p<0.05, ***p<0.001) (n=5-8). There was an effect of sex (p=0.04) and diabetes (p=0.0004) and an interaction between sex*diabetes (p=0.04). (d) Glomerular volume was significantly increased in all diabetic mice compared to their controls, with diabetic male mice having greater glomerular volume compared to female diabetic mice (**p<0.01, ***p<0.001, ****p<0.0001). There was an effect of sex (p<0.0001) and diabetes (p<0.0001). (e-g) Fibrosis was assessed by PSR and IHC for fibronectin (FN) and collagen IV (Col IVα1). Significant increases in all of these were seen for diabetic groups for both males and females, with no effect of sex (**p<0.01, ****p<0.0001).
