# Supplementary material for: Both sexes develop DKD in the CD1 uninephrectomized streptozotocin mouse model
Source: Sci Rep. 2023 Oct 3;13:16635. doi: 10.1038/s41598-023-42670-5 (PMC10547794; doi:10.1038/s41598-023-42670-5)
Supplement: Supplementary file 2 — Supplementary Figure 2. [file 41598_2023_42670_MOESM2_ESM.pdf]

Final Blots Used in Figure 3b

B

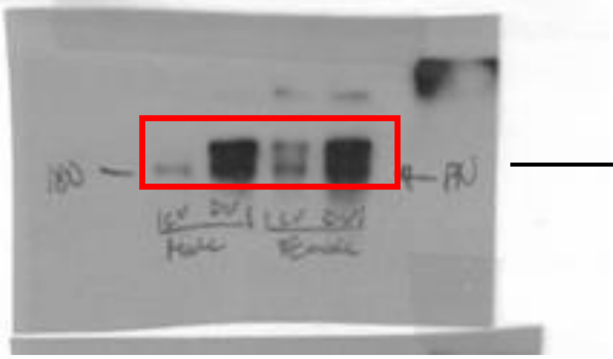

Figure 3b

Representative final figure samples run in blot to the left

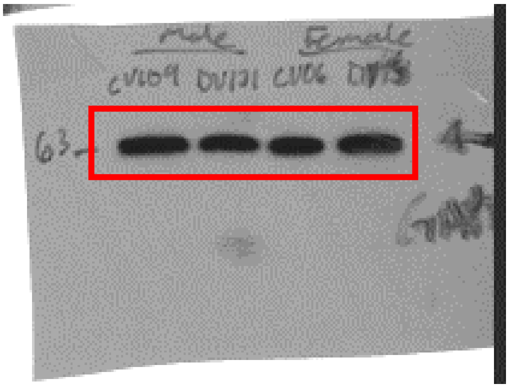

Figure 3b

Representative final figure samples run in blot to the left

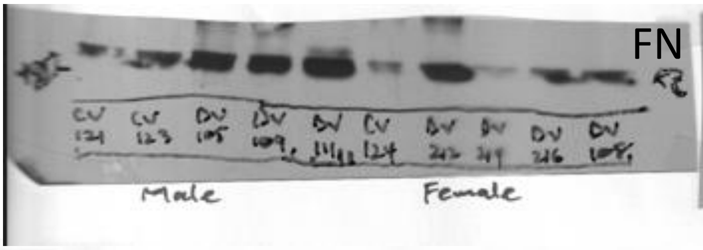

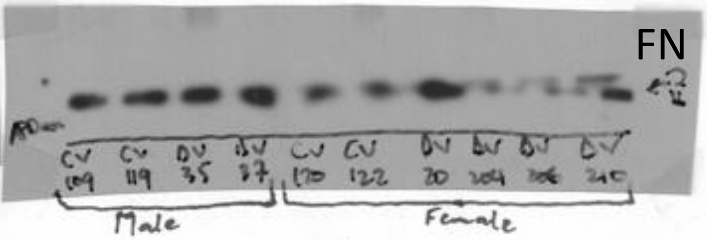

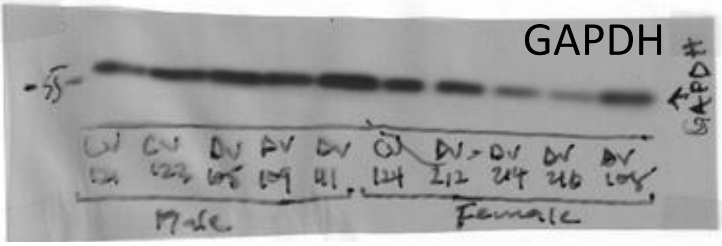

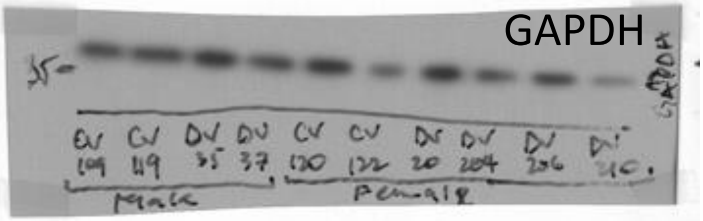

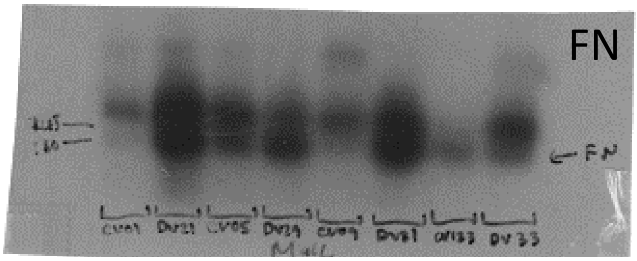

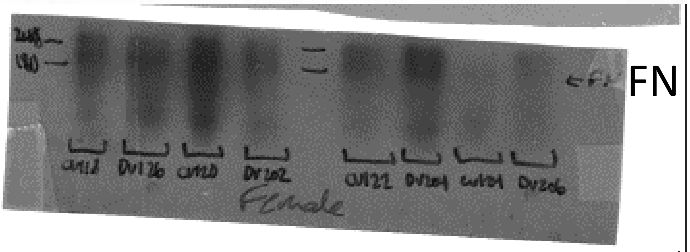

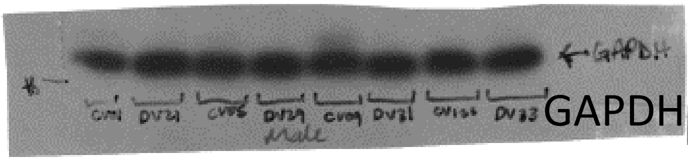

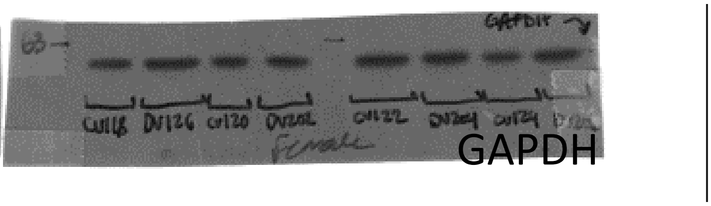

Final Blots Used in Figure 3c

C

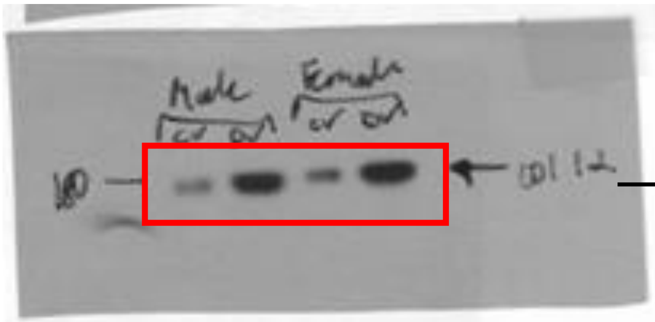

→ Figure 3c

Representative final figure samples run in blot to the left

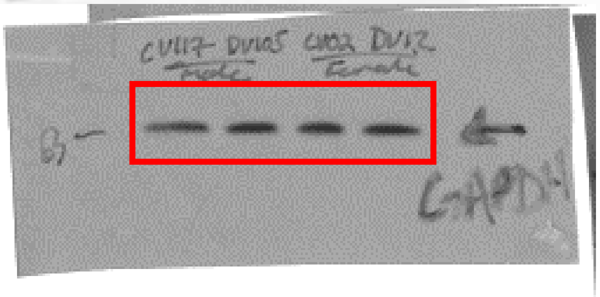

→ Figure 3c

Representative final figure samples run in blot to the left

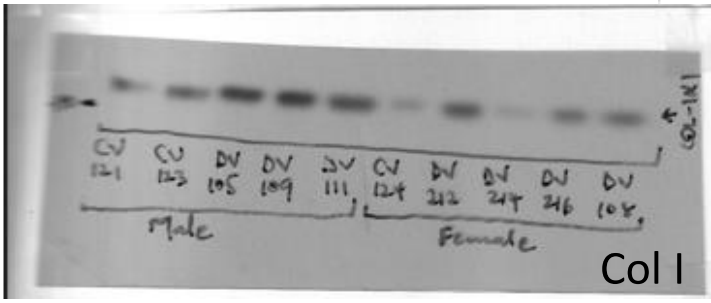

Col I

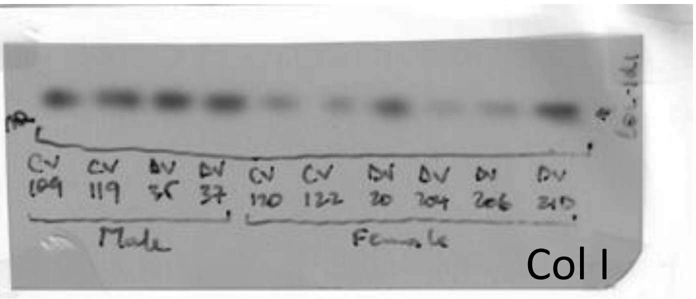

Col I

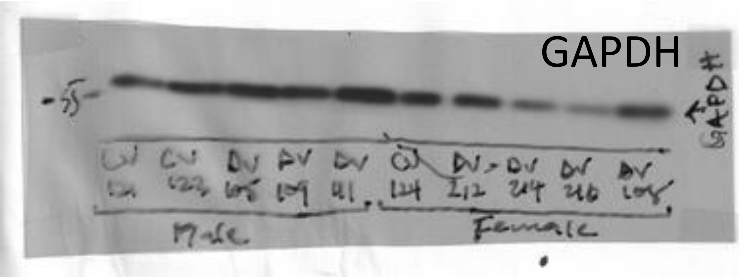

GAPDH

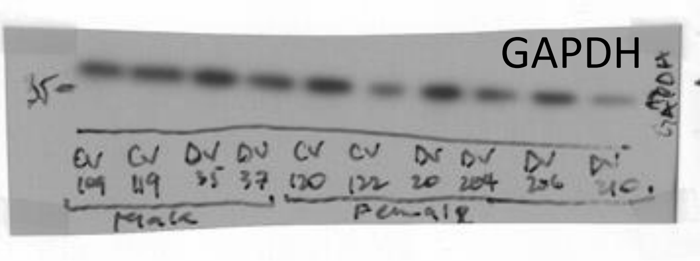

GAPDH

Col I

Col I

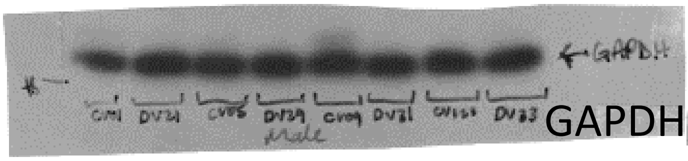

GAPDH

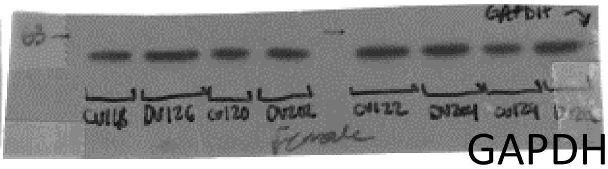

GAPDH
